# Preoperative exercise-based prehabilitation for reducing postoperative pulmonary complications after lung cancer surgery: a systematic review and meta-analysis

**DOI:** 10.3389/fmed.2026.1899789

**Published:** 2026-08-12

**Authors:** Qi-Qi Chen, Yin Liang, YiHong Xu, Lin-Fang Zhao

**Affiliations:** Sir Run Run Shaw Hospital (SRRSH), Zhejiang University School of Medicine, Hangzhou, China

**Keywords:** exercise training, lung cancer, lung resection, meta-analysis, postoperative pulmonary complications, prehabilitation, pulmonary rehabilitation, systematic review

## Abstract

**Background:**

Postoperative pulmonary complications (PPCs) are common and clinically important adverse outcomes after lung cancer surgery. Preoperative exercise-based prehabilitation may enhance functional reserve before surgery, but its effects on PPCs and related perioperative outcomes remain uncertain.

**Methods:**

This systematic review and meta-analysis followed the PRISMA 2020 guidelines and was registered with PROSPERO (CRD420251273259). PubMed, Web of Science, Embase, the Cochrane Library, CINAHL, CNKI, Wanfang Data, and the VIP Database were searched from inception to 25 December 2025. Randomized controlled trials evaluating preoperative exercise-based prehabilitation in adults undergoing lung cancer surgery were included. Risk of bias was assessed using the Cochrane RoB 2 tool, and certainty of evidence was assessed using the GRADE approach. Random-effects meta-analyses were performed using RevMan 5.4.1.

**Results:**

A total of 28 reports comprising 29 randomized controlled trials and 2,900 eligible surgical participants were included. Compared with usual perioperative care, preoperative exercise-based prehabilitation significantly reduced the risk of overall PPCs (RR = 0.42, 95% CI 0.33–0.53; I^2^ = 0%), pneumonia or pulmonary infection (RR = 0.52, 95% CI 0.36–0.75; I^2^ = 0%), atelectasis (RR = 0.36, 95% CI 0.25–0.51; I^2^ = 0%), and respiratory failure (RR = 0.37, 95% CI 0.17–0.78; I^2^ = 0%). Prehabilitation also reduced postoperative length of hospital stay (MD = −2.22 days, 95% CI −2.52 to −1.92; I^2^ = 52%). Results for preoperative 6-minute walk distance were inconsistent and were summarized narratively because of substantial heterogeneity.

**Conclusion:**

Preoperative exercise-based prehabilitation may reduce PPCs and shorten postoperative hospital stay after lung cancer surgery, but the certainty of evidence ranged from moderate to very low across outcomes. Evidence for functional improvement remains uncertain. Future multicenter trials should standardize prehabilitation protocols, outcome definitions, safety monitoring, and adherence reporting.

**Systematic review registration:**

https://www.crd.york.ac.uk/prospero/, identifier CRD420251273259.

## 1 Introduction

Lung cancer remains one of the most common and lethal cancers worldwide. In 2022, approximately 2.5 million new cases and 1.8 million deaths were reported globally, making lung cancer the leading cause of cancer-related mortality (1). For patients with resectable lung cancer, anatomic pulmonary resection remains a cornerstone of curative treatment. Although minimally invasive techniques such as video-assisted thoracoscopic surgery have become increasingly common, postoperative pulmonary complications (PPCs), including pneumonia, atelectasis, and respiratory failure, remain among the most frequent and serious complications after lung cancer surgery (2, 3). Previous studies have reported PPC rates of 20%–30% after lung cancer surgery, and these complications are associated with longer hospital stay, higher mortality, and greater healthcare resource use (3, 4). PPCs are also associated with significantly poorer long-term survival, suggesting that their effects extend beyond immediate postoperative recovery (5).

Patients undergoing lung cancer surgery often have a history of smoking, chronic respiratory disease, and low physical activity, all of which can impair preoperative cardiopulmonary reserve. Chronic obstructive pulmonary disease (COPD), smoking, and reduced pulmonary function are key risk factors for PPCs (6). Because some of these risk factors are modifiable, the preoperative period provides an important opportunity to optimize physiological reserve and reduce perioperative risk. Preoperative prehabilitation is a perioperative strategy centered on exercise training and may also include respiratory training, nutritional support, and psychological interventions. By improving preoperative functional status and physiological reserve, prehabilitation may increase patients’ tolerance of surgical stress and support postoperative recovery (7–9). Existing evidence suggests that preoperative exercise or respiratory training can improve pulmonary function and exercise tolerance in patients with lung cancer and may reduce postoperative complications (10, 11). However, studies vary substantially in intervention components, training intensity, duration, and outcome measures, resulting in inconsistent findings regarding the effect of prehabilitation on PPCs (12). Moreover, some previous systematic reviews combined preoperative and postoperative rehabilitation in a single analysis, which may have obscured the independent effect of preoperative prehabilitation and complicated assessment of its efficacy (13, 14). Therefore, a systematic review and meta-analysis of randomized controlled trials is needed to clarify the effect of preoperative exercise-based prehabilitation on PPCs and related clinical outcomes in patients undergoing lung cancer surgery.

## 2 Materials and methods

### 2.1 Protocol, registration, and reporting guidelines

This systematic review and meta-analysis was conducted and reported in accordance with the Preferred Reporting Items for Systematic Reviews and Meta-Analyses (PRISMA) 2020 statement (15). The PRISMA 2020 checklist and full search strategies are provided in Supplementary Tables 1, 2, respectively. The protocol was registered with PROSPERO (CRD420251273259).

### 2.2 Research question

The review question was formulated using the PICO framework: among adults undergoing lung cancer surgery (P), does preoperative exercise-based prehabilitation, either unimodal or multimodal (I), compared with usual care or standard perioperative management (C), reduce postoperative pulmonary complications (PPCs) and other adverse perioperative outcomes and improve functional recovery (O)?

### 2.3 Eligibility criteria

Eligibility criteria were defined using the PICOS framework. The population comprised adults aged 18 years or older with primary lung cancer, including non-small cell lung cancer and other lung malignancies, who were scheduled for anatomic pulmonary resection. The intervention was preoperative prehabilitation with a structured exercise component, such as aerobic training, resistance training, or respiratory training. Eligible interventions included unimodal and multimodal exercise-focused prehabilitation programs. The comparator was usual care or standard perioperative care. The primary outcome was the overall incidence of PPCs within 30 days after surgery or during the index hospitalization. Secondary outcomes included pneumonia or pulmonary infection, atelectasis, and respiratory failure, each assessed within 30 days after surgery or during hospitalization, as well as length of hospital stay (LOS) and 6-minute walk distance (6MWD). Eligible study designs were randomized controlled trials (RCTs).

Studies were excluded if they met any of the following criteria: non-randomized design; no clearly defined preoperative intervention component or inability to distinguish the effects of preoperative rehabilitation from those of postoperative rehabilitation; duplicate publication; or non-original or non-full-text publication, such as a study protocol, review, or conference abstract. Studies reporting outcomes unsuitable for quantitative meta-analysis were retained and synthesized narratively.

### 2.4 Search strategy

A comprehensive literature search was conducted in PubMed, Web of Science, Embase, the Cochrane Library, CINAHL, China National Knowledge Infrastructure (CNKI), Wanfang Data, and the VIP Database from inception to 25 December 2025. No restrictions were applied by language or publication year. The reference lists of relevant systematic reviews and included studies were also manually screened to identify additional eligible studies.

The PubMed search strategy was initially developed using a combination of controlled vocabulary terms (e.g., MeSH terms) and free-text keywords. It was then adapted for each database, incorporating Emtree terms for Embase and relevant Chinese terms for Chinese databases. Search terms covered three core concepts: lung cancer, lung resection or thoracic surgery, and prehabilitation or exercise-based training. The complete search strategies for all databases are provided in Supplementary Table 2.

### 2.5 Study selection and data extraction

After duplicate records were removed, QQC and YL independently screened the titles and abstracts of all retrieved records and then independently assessed the full texts of potentially eligible studies. Disagreements were resolved through discussion; when consensus could not be reached, LFZ was consulted as a third reviewer.

QQC and YL independently extracted data using a predesigned data extraction form. Extracted data were cross-checked for accuracy and consistency, and any discrepancies were resolved by discussion, with LFZ consulted when necessary. To ensure consistent linkage across the study characteristics table, risk-of-bias assessment, and meta-analysis datasets, each study was assigned a unique identifier. When multiple reports appeared to describe the same trial, on the basis of factors such as the registration number, research team, study setting, and intervention protocol, these reports were merged into a single study record. For the same outcome at the same time point, data were extracted only once. The primary publication or the report with the most complete data was prioritized, and other reports were used to supplement information on trial characteristics or subgroup data to avoid double counting.

### 2.6 Risk-of-bias assessment

Risk of bias in the included RCTs was assessed using the revised Cochrane risk-of-bias tool for randomized trials (RoB 2) (16). QQC and YHX independently performed the assessment across five domains: bias arising from the randomization process, bias due to deviations from intended interventions, bias due to missing outcome data, bias in measurement of the outcome, and bias in selection of the reported result. Overall risk of bias was categorized as “low risk,” “some concerns,” or “high risk.” Disagreements were resolved through discussion, with LFZ consulted as a third reviewer when consensus could not be reached.

### 2.7 Statistical analysis

Quantitative synthesis was performed using Review Manager (RevMan) version 5.4.1. For dichotomous outcomes, risk ratios (RRs) with 95% confidence intervals (CIs) were calculated and pooled using the Mantel–Haenszel method. For continuous outcomes, mean differences (MDs) with 95% CIs were calculated and pooled using the inverse-variance method.

Statistical heterogeneity was assessed using the chi-square test based on Cochran’s Q statistic and the I^2^ statistic. I^2^ values of approximately 25%, 50%, and 75% were interpreted as indicating low, moderate, and high heterogeneity, respectively (17). Because clinical and methodological differences in intervention components, duration, and study design were expected, random-effects models using the DerSimonian–Laird method were applied in the main analyses. Outcomes with substantial clinical heterogeneity or inconsistency that precluded meaningful pooling were described narratively; where appropriate, individual study estimates were presented without a pooled summary estimate.

To improve comparability across time points, the latest assessment within 30 days after surgery was preferentially selected for complication outcomes; if 30-day data were unavailable, in-hospital outcomes were used. For functional outcomes, the 6MWD measured at the end of prehabilitation, specifically the preoperative assessment closest to surgery, was preferentially selected. Multi-arm trials were handled at the trial-outcome level according to the following decision rules. When a trial included more than one eligible preoperative exercise-based prehabilitation arm sharing the same comparator and reporting the same outcome, eligible intervention arms were combined into a single prehabilitation group for the primary meta-analysis. For dichotomous outcomes, event counts and sample sizes were summed. For continuous outcomes, means and standard deviations were combined using standard Cochrane methods based on group sample sizes, means, and standard deviations. Arms that did not meet the eligibility criteria, such as non-surgical groups, postoperative-only rehabilitation groups, or non-exercise/usual-care variants, were excluded from quantitative synthesis. When clinically distinct eligible intervention arms were retained as separate comparisons, the shared control group was split according to RevMan procedures to avoid double counting. The handling of each multi-arm trial and outcome is detailed in Supplementary Table 4.

For rare events, studies with zero events in both arms could not be used to estimate RRs in RevMan. When only one cell contained zero events, RevMan’s default continuity correction was applied. Sensitivity analyses included comparisons between fixed-effect and primary random-effects models and leave-one-out analyses in which one study was removed at a time. For outcomes with at least 10 studies, potential publication bias or small-study effects were assessed by visual inspection of funnel plots and Egger’s regression test. Egger’s regression test was implemented outside RevMan using Python 3.12.13 with NumPy 2.3.5 as an ordinary least-squares regression of the standard normal deviate of the effect estimate against its precision. Log risk ratios and corresponding standard errors were used for dichotomous outcomes, and mean differences and corresponding standard errors were used for continuous outcomes; *P*-values were derived from the t distribution with k - 2 degrees of freedom, where k denotes the number of studies. A two-sided *P*-value < 0.10 was considered suggestive of small-study effects. For outcomes with fewer than 10 studies or outcomes that were not quantitatively pooled, publication bias was assessed qualitatively by considering the number and size of studies, event frequency, risk-of-bias concerns, selective outcome reporting, and the geographical distribution of the available evidence. A two-sided α level of 0.05 was considered statistically significant for the main effect estimates.

### 2.8 Certainty of evidence assessment

The certainty of evidence for each outcome was assessed using the Grading of Recommendations Assessment, Development and Evaluation (GRADE) approach. QQC and YHX independently assessed the certainty of evidence across five domains: risk of bias, inconsistency, indirectness, imprecision, and publication bias. Evidence from randomized controlled trials was initially rated as high certainty and was downgraded by one or two levels when serious or very serious concerns were identified. The final certainty ratings in this review were categorized as high, moderate, low, or very low. Disagreements were resolved through discussion, with LFZ consulted when necessary. A summary of findings table is presented in Table 2, with detailed judgments provided in Supplementary Table 6.

## 3 Results

### 3.1 Study selection

A total of 1,367 records were identified through database searches. After removal of 197 duplicates and two records identified by automated tools as ineligible, 1,168 records remained for title and abstract screening. Of these, 994 records were excluded. A total of 174 reports were sought for retrieval, but two could not be obtained.

**FIGURE 1:**
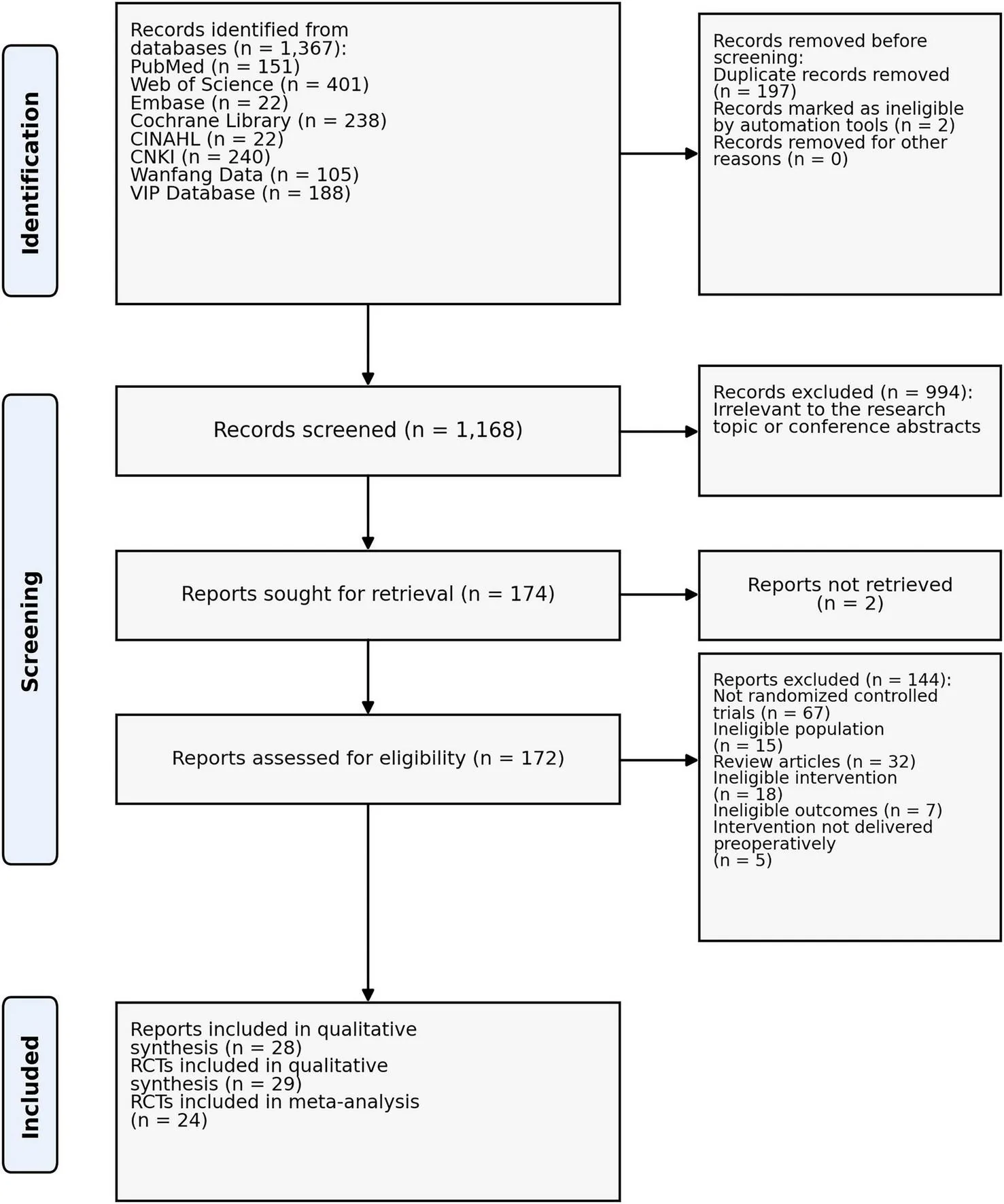
Preferred Reporting Items for Systematic Reviews and Meta-Analyses (PRISMA) 2020 flow diagram of study selection.

After full-text assessment of 172 reports, 144 were excluded because they did not meet the eligibility criteria or for other reasons. For duplicate publications reporting the same trial, only the report with the most complete information was retained. Ultimately, 28 reports covering 29 RCTs were included in the qualitative synthesis, and 24 trials contributed data to at least one outcome in the meta-analysis. The study selection process is shown in the PRISMA 2020 flow diagram (Figure 1).

### 3.2 Characteristics of the included studies

The 28 included articles, reporting 29 RCTs, were published between 2011 and 2025 and involved 2,900 eligible surgical participants, including 1,521 in the intervention groups and 1,379 in the control groups. Most trials were conducted in China (22/29), whereas the remaining trials were conducted in the United States, Switzerland, Portugal, Canada, Spain, or other countries or regions. Most studies were single-center trials (26/29). Individual trial sample sizes ranged from 9 to 320 participants, with a median of approximately 90.

The mean age of participants ranged from 56.2 to 80.8 years, and the proportion of male participants ranged from approximately 31.5%–100%. Most participants had suspected or confirmed lung cancer, primarily non-small cell lung cancer (NSCLC), and were scheduled for pulmonary resection. The most common procedures were lobectomy and segmentectomy. Nine trials specifically enrolled high-risk groups, such as patients with chronic obstructive pulmonary disease (COPD), impaired pulmonary function, or low baseline lung function.

All interventions involved preoperative exercise-based prehabilitation programs. Respiratory training and aerobic exercise were the most frequently used components, followed by resistance training and device-based inspiratory muscle training. Most studies also included behavioral support, such as health education, follow-up, or training logs. Interventions were delivered mainly in hospital settings, although home-based, outpatient, center-based, and hybrid models were also used. The main characteristics of the included studies are summarized in Table 1.

**TABLE 1:** Characteristics of the included studies (*n* = 29).

References	Year	Country	Design	Sample size (I/C)	Intervention components	Duration before surgery	Supervision/monitoring	Setting	Outcomes
Fan et al. (18)	2024	China	RCT	40/40	AER + RESP + RES	7–10 days	Supervised	Healthcare facility	b, d, h, i, l
Gao et al. (19)	2023	China	RCT	120/60	RESP + RES + EDU	≤7 days	Partly supervised: initial trainer guidance followed by home-based prompting	Home and healthcare facility	h, i, l
Chi et al. (20)	2019	China	RCT	33/33	AER + RESP + RES + EDU	≤7 days	Supervised one-to-one training	Healthcare facility	a, b, d, f
Huang et al. (21)	2025	China	RCT	60/30	AER + RESP + HIIT + EDU	≤7 days	Supervised in-hospital training	Healthcare facility	b, h, i
Miao et al. (22)	2020	China	RCT	80/80	AER + RESP + RES + EDU	NR (approximately 1 week mentioned in discussion)	Supervised nurse-led training, including assessment by a specialist rehabilitation nurse	Healthcare facility	a, h, i
Han et al. (23)	2022	China	RCT	69/61	RESP + RES + EDU	≤7 days	Supervised outpatient one-to-one teaching, followed by WeChat official account/telephone reminders and feedback	Home	h, i, k
Su et al. (24)	2020	China	RCT	160/160	RESP + EDU	≤7 days (short in-hospital program)	Supervised; nurses provided one-to-one guidance and monitored training completion after instruction	Healthcare facility	a, b, h, i, n
Wang et al. (25)	2024	China	RCT	40/40	RESP + PSY + EDU	≤7 days	Supervised one-to-one guidance and monitoring by responsible nurses	Healthcare facility	a, b, h
Wu et al. (26)	2018	China	RCT	30/30	AER + RESP + RES + EDU	≤7 days	Supervised by healthcare professionals, with group training plus one-to-one guidance	Healthcare facility	a, b, i
Xue et al. (27)	2023	China	RCT	74/74	AER + RESP + NUT + PSY + EDU	≤14 days (approximately 1 week)	Supervised specialist nurse-led program with family supervision, peer support, and WeChat check-ins	Healthcare facility	a, b, e, g, h
Benzo et al. (28)	2011	United States	RCT	5/4	AER + RESP + RES + EDU	>14 days	Supervised pulmonary rehabilitation (details NR)	Healthcare facility	NR
Benzo et al. (28)	2011	United States	RCT	10/9	AER + RESP + RES + NUT + EDU	≤7 days	Supervised face-to-face sessions	Healthcare facility	a, h, l
Hu et al. (29)	2025	China	RCT	45/47	AER + RESP + RES + EDU	>14 days	Medical staff accompanied stair-training sessions; supervised training	Healthcare facility	a, b, d, h
Licker et al. (30)	2017	Switzerland	RCT	81/83	AER + RES + HIIT + NUT + EDU	>14 days	Supervised outpatient sessions delivered by physiotherapists	Healthcare facility	h
Machado et al. (31)	2024	Portugal	RCT	23/23	AER + RES + EDU	>14 days	Weekly telephone supervision by a physiotherapist	Home	b, c, d, f, m
Patel et al. (32)	2023	Canada	RCT	51/51	AER + RESP + EDU	>14 days	Remote monitoring by the research team with device reminders	Home	h, i, l, m
Su et al. (33)	2025	China	RCT	88/44	RESP + PSY + EDU	7–14 days	NR for direct supervision; participants were trained and device-guided, with WeChat group support, daily logs, and guided videos	Home	a, h, i, l
Yao et al. (34)	2023	China	RCT	74/74	AER + RESP + NUT + PSY + EDU	NR	Nursing-led multidisciplinary team involving a head nurse, nursing supervisors, responsible nurses, and a physician-issued exercise prescription	Healthcare facility	h
Fang et al. (35)	2013	China	RCT	22/22*	AER + RESP + EDU	7–14 days	Supervised cycle ergometry sessions with ECG, BP, and SpO2 monitoring and supplemental oxygen as needed	Healthcare facility	h
Geng et al. (36)	2019	China	RCT	20/20	AER + RESP + NUT + EDU	7–14 days	In-hospital guided training; some components were device- or feedback-assisted	Healthcare facility	a, b, h
Li et al. (37)	2023	China	RCT	25/25	AER + RESP + NUT + EDU	≤7 days	Supervised or guided in-hospital training	Healthcare facility	a, b, h
Liu et al. (38)	2024	China	RCT	44/44	AER + RESP + RES + NUT + PSY + EDU	15–30 days	Multidisciplinary prehabilitation team led by a nurse manager, with surgeons, a geriatrician, dietitian, rehabilitation therapist, psychologist, and specialist nurses	Healthcare facility	a, b, c, e, g, h, l
Li et al. (39)	2024	China	RCT	49/49	AER + RESP + RES + NUT + PSY + EDU	7–14 days	Remote, education-supported home program; family assistance for stair training	Home	a, e, h, i, l
Zhang et al. (40)	2014	China	RCT	43/43	AER + RESP + EDU	7–14 days	Nurse-supervised sessions	Healthcare facility	a, b, h
Yang et al. (41)	2022	China	RCT	47/44	AER + RESP + PSY + EDU	15–28 days	Daily telephone/WeChat follow-up by prehabilitation nurses, rehabilitation therapist involvement, and family assistance for older adults	Home and healthcare facility	a, b, e, h, i, l, n
Bhatia et al. (42)	2019	Switzerland	RCT	74/77	AER + HIIT + NUT + EDU	15–28 days	Supervised by experienced respiratory physiotherapists (1–3 patients per session)	Healthcare facility	a, b
Lai et al. (43)	2017	China	RCT	51/50	AER + RESP + EDU	≤7 days	Training delivered by nurses and physiotherapists/rehabilitation therapists, with monitoring during training	Healthcare facility	a, b, d, f, g, h, k
Sebio García et al. (44)	2017	Spain	RCT	20/20	AER + RESP + RES + NUT + EDU	>14 days	Supervised outpatient sessions delivered by physiotherapists; home spirometry was self-administered	Home and healthcare facility	a, b, c, d, f, h, i
Liu et al. (45)	2020	China	RCT	43/42	AER + RESP + RES + NUT + PSY + EDU	>14 days	Home-based program with instruction booklet and weekly telephone calls; resistance training was demonstrated and corrected at baseline visit	Home	b, h, i, l

Study identifiers correspond to the reference numbers in the manuscript. Intervention components: AER, aerobic exercise; RESP, respiratory training; RES, resistance exercise; HIIT, high-intensity interval training; NUT, nutritional support; PSY, psychological support; EDU, health education. Outcomes: a, pulmonary function; b, physical activity or exercise capacity; c, muscle strength; d, quality of life; e, psychological status; f, body composition; g, nutritional indicators; h, postoperative complications; i, length of hospital stay; j, 30-day readmission; k, hospitalization costs; l, postoperative chest-tube duration; m, feasibility and safety; n, adherence. RCT, randomized controlled trial; I/C, intervention/control; NR, not reported; ECG, electrocardiography; BP, blood pressure; SpO2, peripheral oxygen saturation; PR, pulmonary rehabilitation.

*For Fang et al., I/C reflects the operable randomized surgical comparison (22/22); 17 non-operative participants receiving training were excluded from quantitative synthesis.

### 3.3 Risk-of-bias assessment

**FIGURE 2:**
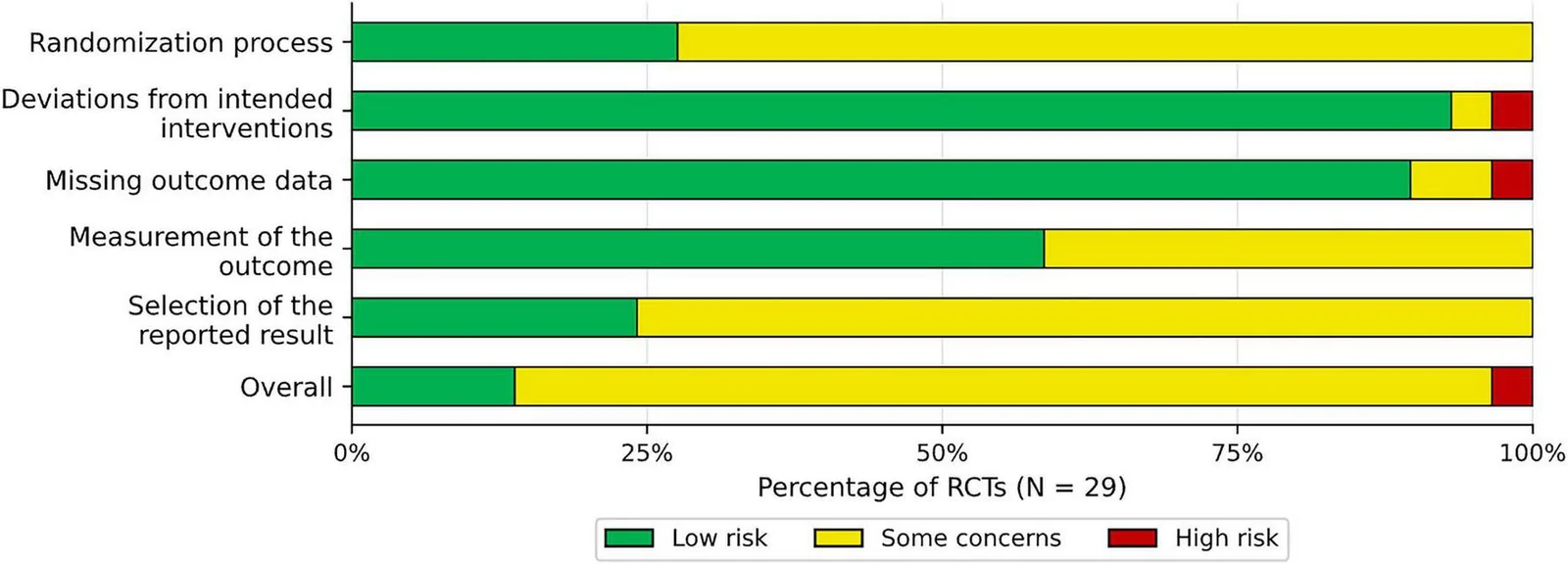
Risk-of-bias summary based on the RoB 2 tool.

**FIGURE 3:**
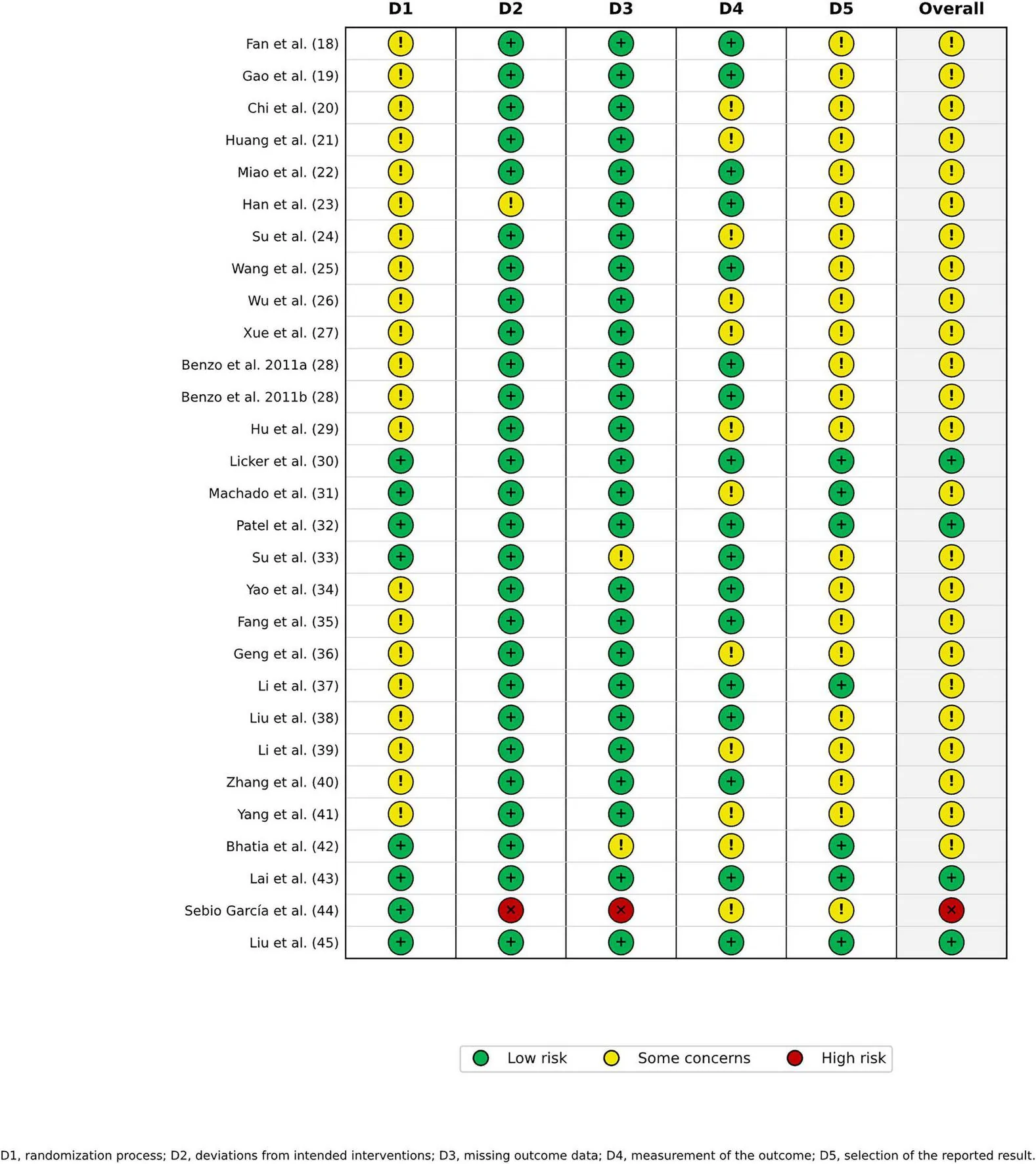
Traffic-light plot of risk-of-bias judgments based on the RoB 2 tool. D1, randomization process; D2, deviations from intended interventions; D3, missing outcome data; D4, measurement of the outcome; D5, selection of the reported result. The two trials reported by Benzo et al. were evaluated separately.

The RoB 2 assessment was performed for the 29 included RCTs. Overall risk of bias was judged as low in 4 RCTs (13.8%), as having some concerns in 24 RCTs (82.8%), and as high in 1 RCT (3.4%). The most common concerns involved the randomization process (21/29) and selection of the reported result (22/29), mainly because allocation concealment and prospective reporting details were not adequately described. Deviations from intended interventions and missing outcome data were rated as low risk in most RCTs (27/29 and 26/29, respectively). Measurement of outcomes was rated as low risk in 17 RCTs and as having some concerns in 12 RCTs, largely because blinding of outcome assessors was not consistently documented. One RCT was rated as having a high overall risk of bias, mainly because of high-risk judgments for deviations from intended interventions and missing outcome data. The risk-of-bias summary and traffic-light plot are shown in Figures 2, 3.

### 3.4 Meta-analysis

#### 3.4.1 Primary outcome: overall PPCs within 30 days or during hospitalization

**FIGURE 4:**
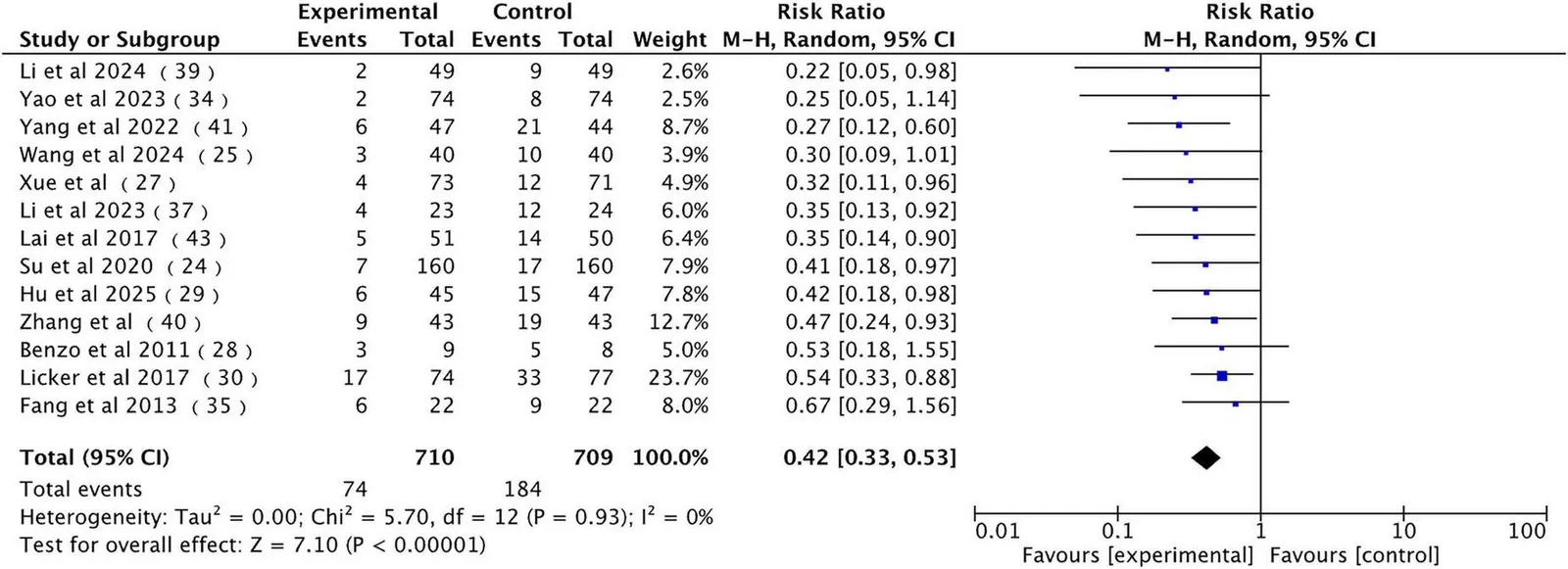
Effects of preoperative exercise-based prehabilitation on overall postoperative pulmonary complications (PPCs).

Thirteen studies were included in the meta-analysis of overall PPCs. Compared with the control group, preoperative exercise-based prehabilitation significantly reduced the risk of overall PPCs, with no observed statistical heterogeneity across studies (RR = 0.42, 95% CI 0.33–0.53; I^2^ = 0%; Figure 4). In absolute terms, PPCs occurred in 74 of 710 participants (10.4%) in the intervention group and 184 of 709 participants (26.0%) in the control group.

#### 3.4.2 Secondary outcome: pneumonia or pulmonary infection within 30 days or during hospitalization

**FIGURE 5:**
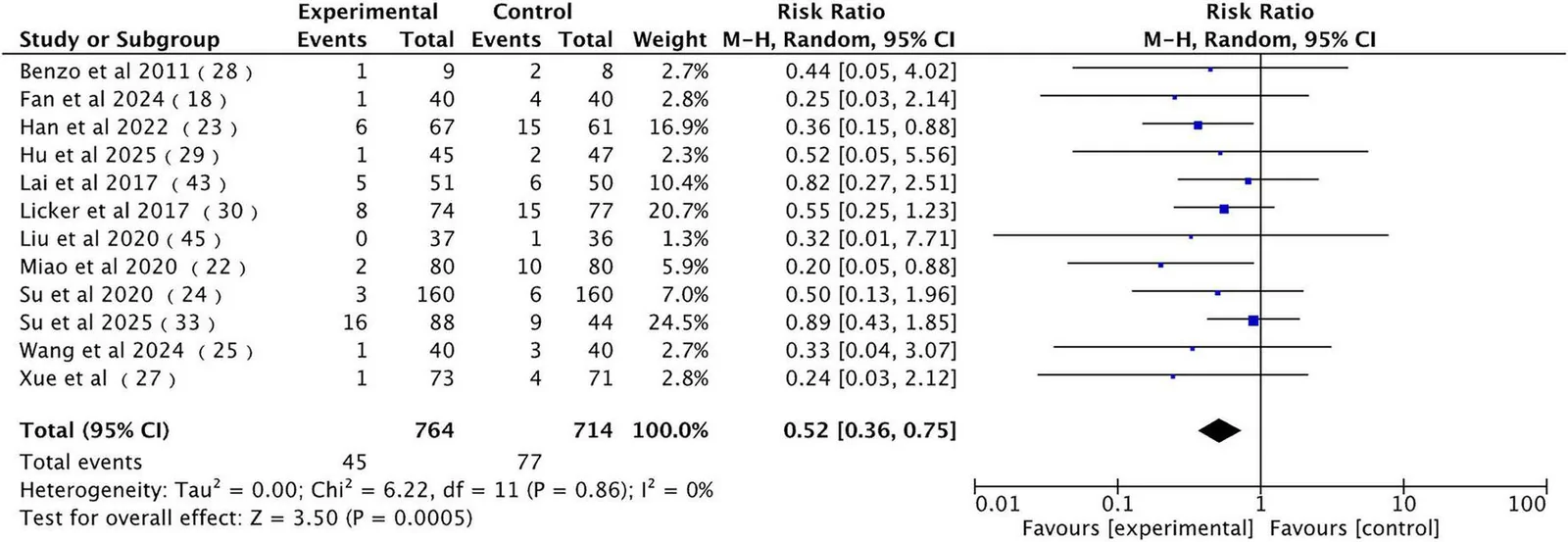
Effects of preoperative exercise-based prehabilitation on pneumonia or pulmonary infection.

Twelve studies were included in the meta-analysis of pneumonia or pulmonary infection. Preoperative exercise-based prehabilitation reduced the risk of pneumonia or pulmonary infection compared with the control intervention, with no observed statistical heterogeneity (RR = 0.52, 95% CI 0.36–0.75; I^2^ = 0%; Figure 5). The event rate was 45 of 764 participants (5.9%) in the intervention group and 77 of 714 participants (10.8%) in the control group.

#### 3.4.3 Secondary outcome: atelectasis within 30 days or during hospitalization

**FIGURE 6:**
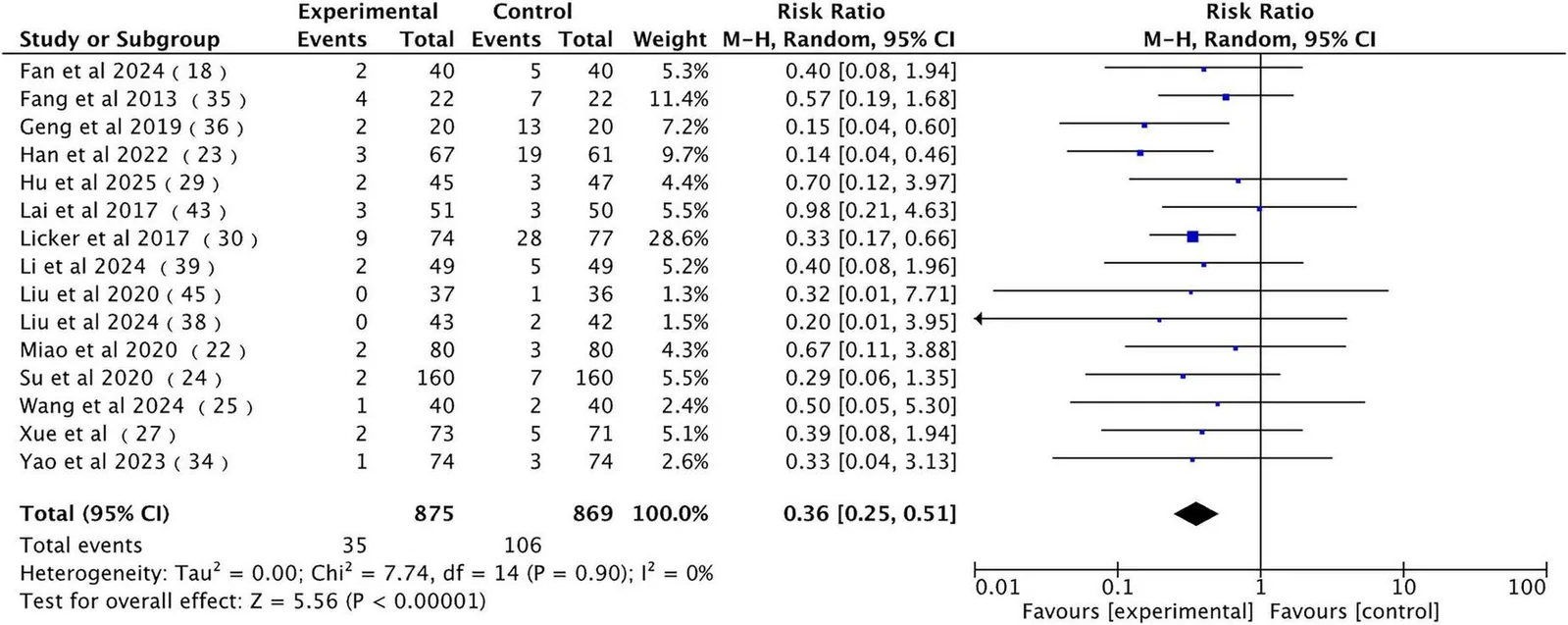
Forest plot of the effects of preoperative exercise-based prehabilitation on atelectasis.

Fifteen studies were included in the meta-analysis of atelectasis. Preoperative exercise-based prehabilitation reduced the risk of atelectasis compared with the control intervention, with no observed statistical heterogeneity (RR = 0.36, 95% CI 0.25–0.51; I^2^ = 0%; Figure 6). The event rate was 35 of 875 participants (4.0%) in the intervention group and 106 of 869 participants (12.2%) in the control group.

#### 3.4.4 Secondary outcome: respiratory failure within 30 days or during hospitalization

**FIGURE 7:**
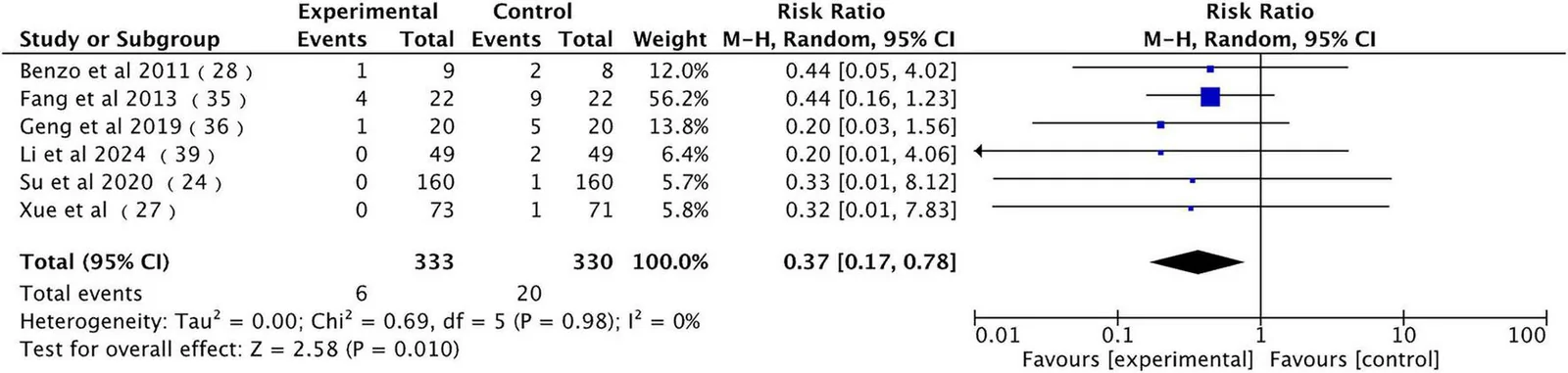
Effects of preoperative exercise-based prehabilitation on respiratory failure.

Six studies were included in the meta-analysis of respiratory failure. Preoperative exercise-based prehabilitation reduced the risk of respiratory failure compared with the control intervention, with no observed statistical heterogeneity (RR = 0.37, 95% CI 0.17–0.78; I^2^ = 0%; Figure 7). The event rate was 6 of 333 participants (1.8%) in the intervention group and 20 of 330 participants (6.1%) in the control group.

#### 3.4.5 Secondary outcome: length of hospital stay

**FIGURE 8:**
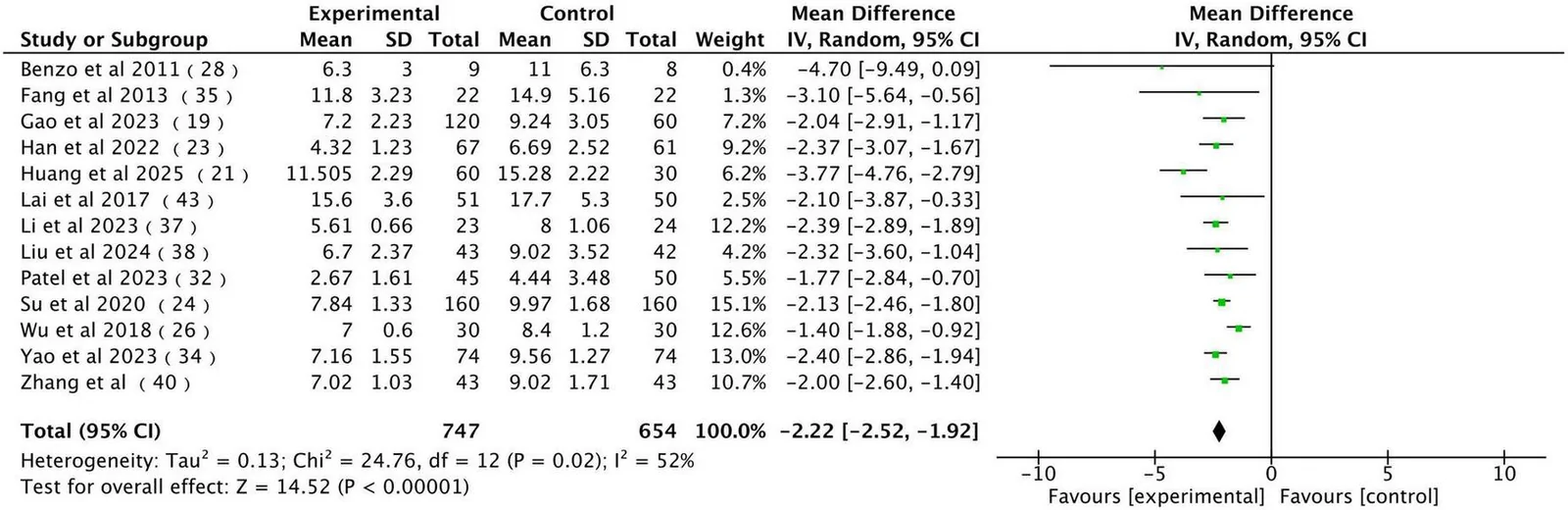
Effects of preoperative exercise-based prehabilitation on length of hospital stay.

Thirteen studies were included in the meta-analysis of length of hospital stay (LOS). Preoperative exercise-based prehabilitation reduced postoperative LOS compared with the control intervention (MD = -2.22 days, 95% CI −2.52 to −1.92), although moderate heterogeneity was observed across studies (I^2^ = 52%; Figure 8).

#### 3.4.6 Secondary outcome: 6-minute walk distance

Four studies reported preoperative 6-minute walk distance (6MWD). During the initial assessment for quantitative synthesis, substantial statistical heterogeneity was observed (I^2^ = 95%), along with notable clinical differences in intervention protocols, training duration, and baseline participant characteristics. Therefore, a pooled effect estimate was not considered clinically appropriate, and this outcome was summarized narratively.

The available evidence showed mixed effects of preoperative exercise-based prehabilitation on preoperative 6MWD. Three studies reported greater preoperative 6MWD in the prehabilitation group than in the control group, with mean differences ranging from approximately 49.91 to 84.75 m. In contrast, Lai et al. reported a lower preoperative 6MWD in the prehabilitation group than in the control group. Given the small number of studies, substantial heterogeneity, and divergent direction of effect, the current evidence is insufficient to confirm that preoperative exercise-based prehabilitation consistently improves preoperative 6MWD.

### 3.5 Sensitivity analysis

Sensitivity analyses included comparisons between the primary random-effects models and fixed-effect models and systematic leave-one-out analyses. The direction and statistical significance of the pooled estimates for overall PPCs, pneumonia or pulmonary infection, atelectasis, respiratory failure, and LOS remained largely consistent (Supplementary Table 3). For complication outcomes, fixed-effect estimates were nearly identical to the primary random-effects estimates, and leave-one-out analyses did not materially change the pooled effects. The leave-one-out RR ranges were 0.39–0.44 for overall PPCs, 0.44–0.56 for pneumonia or pulmonary infection, 0.34–0.39 for atelectasis, and 0.28–0.40 for respiratory failure. For LOS, the pooled effect remained statistically significant after each study was removed individually (leave-one-out MD range: −2.31 to −2.11 days). Heterogeneity for LOS decreased after exclusion of Huang et al. (I^2^ = 22%), suggesting that this study may have contributed to between-study heterogeneity; however, the pooled effect continued to favor prehabilitation.

### 3.6 Publication bias and small-study effects

Funnel plots and Egger’s regression tests were generated for outcomes with at least 10 studies, including overall PPCs, pneumonia or pulmonary infection, atelectasis, and LOS (Supplementary Figures 1–4 and Supplementary Table 5). Visual inspection suggested possible asymmetry for overall PPCs and LOS, whereas funnel plots for pneumonia or pulmonary infection and atelectasis did not show marked asymmetry. Egger’s regression test suggested possible small-study effects for overall PPCs (*P* = 0.016) and pneumonia or pulmonary infection (*P* = 0.065), but did not provide statistical evidence of small-study effects for atelectasis (*P* = 0.625) or LOS (*P* = 0.285). These findings should be interpreted cautiously because several analyses included sparse events and clinically heterogeneous intervention protocols.

Funnel plots and Egger’s tests were not performed for respiratory failure because fewer than 10 studies were available and the number of events was low. Publication bias for respiratory failure could therefore not be ruled out. For preoperative 6MWD, publication bias could not be formally assessed because only four studies reported this outcome and no pooled estimate was calculated. Qualitatively, reporting bias or small-study effects could not be excluded for these outcomes because the available evidence was limited, several studies were small single-center trials, and the evidence base was geographically clustered, with 22 of 29 RCTs conducted in China and 26 of 29 RCTs conducted at single centers.

### 3.7 Certainty of evidence

The certainty of evidence assessed using the GRADE approach is summarized in Table 2, with detailed judgments provided in Supplementary Table 6. The certainty of evidence ranged from moderate to very low across outcomes. Evidence certainty was downgraded mainly because of risk-of-bias concerns, possible publication bias or small-study effects, sparse events, imprecision, and inconsistency. The certainty of evidence was rated as moderate for atelectasis, low for overall PPCs, pneumonia or pulmonary infection, respiratory failure, and LOS, and very low for preoperative 6MWD. For respiratory failure, the certainty of evidence was limited by sparse events and qualitative concerns regarding publication bias; for preoperative 6MWD, it was further limited by serious inconsistency, imprecision related to the small number of studies and participants, and publication/reporting bias that could not be formally tested.

**TABLE 2:** Summary of findings and certainty of evidence.

Outcome	Effect estimate	Studies/participants	Certainty of evidence	Main reasons for downgrading
Overall PPCs	RR = 0.42, 95% CI 0.33–0.53	13 RCTs/1,419 participants	Low	Risk-of-bias concerns; possible publication bias/small-study effects
Pneumonia or pulmonary infection	RR = 0.52, 95% CI 0.36–0.75	12 RCTs/1,478 participants	Low	Risk-of-bias concerns; possible small-study effects
Atelectasis	RR = 0.36, 95% CI 0.25–0.51	15 RCTs/1,744 participants	Moderate	Risk-of-bias concerns
Respiratory failure	RR = 0.37, 95% CI 0.17–0.78	6 RCTs/663 participants	Low	Risk-of-bias concerns; sparse events/imprecision; publication bias could not be formally tested
Length of hospital stay	MD = −2.22 days, 95% CI −2.52 to −1.92	13 RCTs/1,401 participants	Low	Risk-of-bias concerns; inconsistency; possible context-specific discharge practices
Preoperative 6MWD	Not pooled	4 RCTs/274 participants	Very low	Risk-of-bias concerns; serious inconsistency; imprecision/few studies; publication/reporting bias could not be formally tested

### 3.8 Safety and adherence

Most included studies did not systematically report exercise-related adverse events, adherence measures such as training completion rates or achievement of the prescribed training dose, or reasons for discontinuation using standardized methods. Because reporting approaches varied substantially, these outcomes were not pooled quantitatively.

Two studies reported that no serious exercise-related adverse events occurred during the intervention period, suggesting that preoperative exercise-based prehabilitation is generally safe (31, 32). A few studies also documented good adherence to the training programs, indicating that most participants were able to complete the prescribed training plan with guidance from healthcare professionals, follow-up reminders, or family support (24, 41). However, because safety and adherence were reported inconsistently and without standardized indicators, the current evidence remains limited. Future studies should standardize reporting of these outcomes to enable a more comprehensive assessment of the feasibility and safety of preoperative exercise-based prehabilitation.

## 4 Discussion

### 4.1 Preoperative exercise-based prehabilitation reduces the risk of PPCs and shortens postoperative length of stay in patients with lung cancer

This systematic review and meta-analysis demonstrated that preoperative exercise-based prehabilitation reduces the risk of PPCs and improves postoperative recovery in patients undergoing lung cancer surgery, consistent with previous evidence (46). After lobectomy or segmentectomy, patients with lung cancer may experience respiratory muscle weakness, impaired ventilation, and retention of airway secretions due to anesthesia, surgical trauma, and postoperative pain. These factors can increase the risk of atelectasis, pneumonia, and other pulmonary complications. Previous studies have shown that respiratory muscle training or airway clearance exercises combined with exercise training can strengthen respiratory muscles and promote airway secretion clearance, thereby reducing PPCs (19, 36). In addition, some studies incorporated health education, nutritional support, or psychological interventions into exercise-based prehabilitation programs, which may further improve patients’ overall preoperative condition (27, 34, 38, 41).

In the included studies, prehabilitation generally lasted from 5 days to 4 weeks. For example, Gao et al. implemented 5–10 days of respiratory training, whereas Licker et al., Sebio et al. delivered approximately 25 days of high-intensity interval training (19, 30, 44). Although intervention periods were relatively short, the present meta-analysis still observed a significant reduction in postoperative complications. Within the limited preoperative window, exercise training may improve respiratory function and physical reserve, facilitate early postoperative mobilization, and shorten postoperative length of stay. However, intervention models, training intensity, and intervention duration varied substantially across studies. Large-scale, multicenter RCTs are therefore needed to determine the optimal implementation strategy for preoperative prehabilitation.

Sensitivity analyses supported the robustness of the main findings; however, funnel-plot asymmetry for some outcomes suggests that small-study effects or selective reporting may still exist. The results should therefore be interpreted with appropriate caution.

### 4.2 The effect of preoperative exercise-based prehabilitation on functional reserve requires further investigation

Only four RCTs included in this review reported preoperative 6-minute walk distance (6MWD). During exploratory quantitative assessment, substantial heterogeneity was observed across studies (I^2^ = 95%); therefore, a pooled effect was not provided. The findings remain inconsistent. Individual study estimates showed that Geng et al., Li et al., Zhang et al. reported higher preoperative 6MWD in the prehabilitation group, whereas Lai et al. reported lower preoperative 6MWD in the prehabilitation group than in the control group (36, 37, 40, 43). These findings suggest that the effect of preoperative exercise-based prehabilitation on preoperative exercise capacity remains uncertain.

This inconsistency may reflect differences in intervention protocols and duration. Some included studies used relatively short preoperative training programs; for example, Gao et al. implemented approximately 5–10 days of respiratory training, whereas the interventions by Licker et al., Sebio et al., Liu et al. lasted approximately 2–4 weeks (19, 30, 38, 44). Compared with longer and more systematic training programs, short-term interventions may improve respiratory muscle function or exercise tolerance, but their effect on objective measures of functional capacity, such as 6MWD, may be limited. Training models also varied across studies. For example, Licker et al., Sebio et al. used high-intensity interval training, whereas Patel et al. used a home-based, remotely monitored exercise training model (30, 32, 44). These differences may have affected the consistency of functional outcomes. Overall, evidence regarding the effect of preoperative prehabilitation on functional indicators such as 6MWD remains inconsistent, and the effect may depend on intervention duration, training modality, and baseline physical fitness (13). Future studies should standardize the timing of functional outcome assessment and evaluate changes in exercise capacity before and after surgery to better clarify the effect of preoperative prehabilitation on functional reserve and postoperative recovery.

### 4.3 Preoperative exercise-based prehabilitation interventions are heterogeneous in content, delivery, and duration

The preoperative exercise-based prehabilitation interventions included in this review varied in training components, delivery settings, and duration. Most focused on exercise training and were combined with respiratory training, health education, or nutritional support as part of multimodal prehabilitation. Some studies used aerobic training methods, such as walking, cycle ergometry, or cycling, together with respiratory training and evaluated their effects on overall cardiopulmonary endurance (18, 35, 36). Other studies focused on respiratory function training, including diaphragmatic breathing, pursed-lip breathing, respiratory muscle training, and effective coughing, and reported improvements in pulmonary function indicators and reductions in postoperative pulmonary complications (19, 25, 40).

Regarding delivery, interventions mainly included facility-based supervised training, home-based training, and hybrid models combining pre-admission and in-hospital components. For patients with impaired pulmonary function, COPD, or severe deconditioning, Sebio et al. suggested that supervised training delivered by rehabilitation therapists or healthcare professionals in hospital or outpatient settings may allow more precise control of training intensity and better safety monitoring (44). Some studies used home-based training models and enhanced adherence through telephone follow-up or remote guidance (31, 32). Yang et al. developed an ERAS-based continuous prehabilitation pathway that integrated home-based preoperative training with in-hospital training and delivered comprehensive interventions through multidisciplinary collaboration (41).

In terms of intervention duration, the included studies ranged from ultra-short programs lasting 5–7 days to more traditional programs lasting 2–4 weeks, with some studies using longer intervention periods. Ultra-short programs were often implemented while patients awaited surgery during hospitalization (37). Short-term programs of approximately 2 weeks were more common, such as the 2-week respiratory and stair-climbing training program reported by Zhang et al. and the program by Su et al., which combined respiratory training with Liuzijue breathing exercises during a smoking-cessation preparation period of at least 2 weeks (33, 40). Some studies also offered flexible 2–4-week programs tailored to the surgical schedule, extending the intervention by another 2 weeks for patients who did not achieve training goals (41). These variations indicate that the timing of prehabilitation before lung cancer surgery is not merely a methodological inconsistency but is closely linked to the oncological surgical window, patients’ baseline physical condition, risk factors, and access to healthcare resources (47). Therefore, prehabilitation should not be viewed simply as “the longer, the better”; rather, the goal should be to maximize intervention effectiveness within the limited preoperative period without delaying surgery.

Based on these findings, clinical implementation of prehabilitation should be individualized according to patient characteristics, timing of surgery, and risk level, with flexible adjustment of training components, intervention duration, and outcome measures (48). Nurses play a vital role in patient assessment, health education, adherence management, and coordination within multidisciplinary prehabilitation teams. Within the ERAS framework, specialist nurses or nurse navigators should have competencies in perioperative assessment, patient education, rehabilitation guidance, and multidisciplinary coordination (49, 50). Future research should further evaluate nurse navigator-led preoperative prehabilitation models within the ERAS framework and incorporate remote follow-up or digital technologies to improve access and adherence, thereby promoting more consistent implementation of preoperative prehabilitation in perioperative care for patients undergoing lung cancer surgery.

### 4.4 Limitations of the study

Several limitations should be considered when interpreting these findings. First, although this review included only RCTs, many were single-center studies with relatively small sample sizes, and most were conducted in China. This may limit the generalizability of the findings to other healthcare systems, surgical pathways, and rehabilitation settings. Second, interventions varied substantially in training components, intensity, supervision, delivery settings, and duration. As a result, this review could not determine the optimal prehabilitation protocol or the specific contribution of individual components, such as aerobic training, respiratory training, resistance exercise, nutritional support, or psychological support. Third, definitions and assessment of PPCs were not fully standardized across studies. Some studies reported 30-day complications, whereas others reported complications during the index hospitalization, which may have introduced differences in event ascertainment. LOS may also have been influenced by local discharge criteria, ERAS implementation, and healthcare resource availability, rather than by prehabilitation alone.

Fourth, the overall risk of bias was judged as having some concerns, mainly because of insufficient reporting of allocation concealment, blinding of outcome assessors, and prespecified outcome reporting. Fifth, adherence, completion of the prescribed training dose, and exercise-related adverse events were reported inconsistently, preventing a robust quantitative assessment of feasibility and safety. Sixth, substantial heterogeneity was observed for preoperative 6MWD and moderate-to-substantial heterogeneity for LOS; although sensitivity analyses identified a potential source of heterogeneity for LOS, residual clinical and methodological differences remained. Finally, although funnel plots and Egger’s tests were used where appropriate, the possibility of publication bias and small-study effects could not be excluded. This concern was particularly relevant for outcomes with fewer than 10 studies, sparse events, or no quantitative pooling, such as respiratory failure and preoperative 6MWD. In addition, the evidence base was geographically clustered, with 22 of 29 RCTs conducted in China and 26 of 29 RCTs conducted at single centers, which may limit the generalizability of the findings. Although the GRADE approach was applied, the certainty of evidence was limited by risk-of-bias concerns, possible publication bias or small-study effects, sparse events for some outcomes, and substantial inconsistency for LOS and preoperative 6MWD. These limitations indicate that the findings should be interpreted with caution and confirmed in future large-scale, multicenter RCTs with standardized intervention protocols and outcome definitions.

## 5 Conclusion

This systematic review and meta-analysis examined the effects of preoperative exercise-based prehabilitation on perioperative outcomes in patients undergoing lung cancer surgery. The findings suggest that preoperative exercise-based prehabilitation reduces the overall risk of PPCs and lowers the rates of pneumonia or pulmonary infection, atelectasis, and respiratory failure, while also shortening postoperative LOS. However, its effect on preoperative 6MWD remains inconsistent. The certainty of evidence ranged from moderate to very low, indicating that the findings should be interpreted cautiously, particularly for respiratory failure, LOS, and preoperative 6MWD. The included studies varied in intervention components, delivery methods, and duration, suggesting that different prehabilitation models may have different effects on functional outcomes. Future large-scale, multicenter RCTs are needed to establish standardized preoperative prehabilitation protocols and outcome measures, identify more effective intervention strategies, and strengthen the evidence supporting routine use of prehabilitation in perioperative care for patients undergoing lung cancer surgery.

## Data Availability

The original contributions presented in this study are included in the article/Supplementary material, further inquiries can be directed to the corresponding author.
