# Supplementary material for: Preoperative exercise-based prehabilitation for reducing postoperative pulmonary complications after lung cancer surgery: a systematic review and meta-analysis
Source: Front Med (Lausanne). 2026 Aug 12;13:1899789. doi: 10.3389/fmed.2026.1899789 (PMC13506725; doi:10.3389/fmed.2026.1899789)

# Supplementary Material

## Supplementary Table S1. PRISMA 2020 checklist

Note: The checklist has been updated to reflect the finalized manuscript title, structured abstract, publication-bias assessment, and GRADE certainty-of-evidence assessment.

| PRISMA item | Topic | Location in manuscript | Comment |
|---|---|---|---|
| 1 | Title | Title page | The title identifies the work as a systematic review and meta-analysis of randomized controlled trials. |
| 2 | Abstract | Abstract | The structured abstract reports the background, methods, main results, and conclusion without citations. |
| 3 | Rationale | Introduction | Clinical importance of PPCs and the rationale for preoperative exercise-based prehabilitation are described. |
| 4 | Objectives | Introduction; Research question | PICO-based review question is reported. |
| 5 | Eligibility criteria | Materials and methods - Eligibility criteria | PICOS eligibility criteria are specified. |
| 6 | Information sources | Materials and methods - Search strategy | All searched databases and manual reference-list searches are reported. |
| 7 | Search strategy | Materials and methods - Search strategy; Supplementary Table S2 | Complete database-specific search strategies are provided. |
| 8 | Selection process | Materials and methods - Study selection and data extraction | Two-reviewer independent screening and arbitration by a third reviewer are described. |
| 9 | Data collection process | Materials and methods - Study selection and data extraction | Two-reviewer data extraction and duplicate-publication handling are described. |
| 10 | Data items | Eligibility criteria; Study selection and data extraction | Population, intervention, comparator, outcomes, and study design are defined. |
| 11 | Risk-of-bias assessment | Materials and methods - Risk-of-bias assessment | RoB 2 domains and judgment categories are described. |
| 12 | Effect measures | Materials and methods - Statistical analysis | RRs for dichotomous outcomes and MDs for continuous outcomes are specified. |
| 13 | Synthesis methods | Materials and methods - Statistical analysis | Model choice, heterogeneity assessment, handling of zero events, multi-arm trials, narrative synthesis, and sensitivity analyses are described. |
| 14 | Reporting-bias assessment | Materials and methods - Statistical analysis | Funnel plots and Egger's tests were specified for outcomes with at least 10 studies; qualitative assessment was specified for outcomes with fewer than 10 studies or no pooled estimate. |
| 15 | Certainty assessment | Methods, Certainty of evidence assessment; Table 2; Supplementary Table S6 | Certainty of evidence was assessed using the GRADE approach across risk of bias, inconsistency, indirectness, imprecision, and publication bias. |
| 16 | Study selection | Results - Study selection; Figure 1 | Records, reports, exclusions, and included studies are reported in the PRISMA flow diagram. |
| 17 | Study characteristics | Results - Characteristics of the included studies; Table 1 | Characteristics of 28 reports covering 29 RCTs are summarized. |
| 18 | Risk of bias in studies | Results - Risk-of-bias assessment; Figures 2 and 3 | Overall RoB 2 results are summarized. |
| 19 | Results of individual studies | Figures 4-8 | Individual study estimates for pooled outcomes are displayed in forest plots; 6MWD is summarized narratively. |
| 20 | Results of syntheses | Results - Meta-analysis | Pooled estimates are reported for PPCs, pneumonia/pulmonary infection, atelectasis, respiratory failure, and LOS; 6MWD is narratively synthesized. |
| 21 | Reporting biases | Results – Publication bias and small-study effects; Supplementary Figures S1–S4; Supplementary Table S5. | Funnel-plot assessment is reported for outcomes with at least 10 studies. |
| 22 | Certainty of evidence | Results, Certainty of evidence; Table 2; Supplementary Table S6 | Certainty of evidence is summarized for each outcome, with detailed judgments in Supplementary Table S6. |
| 23 | Discussion | Discussion; Limitations of the study | Main findings, interpretation, intervention heterogeneity, clinical implications, and limitations are discussed. |
| 24 | Registration and protocol | Materials and methods - Protocol, registration, and reporting guidelines | PROSPERO registration number is reported. |
| 25 | Support | Funding statement | Funding statement reports no financial support |
| 26 | Competing interests | Conflict of interest | Conflict-of-interest statement is included. |
| 27 | Availability of data, code, and materials | Data availability statement; Supplementary material | Data are available in the article and supplementary material. |

## Supplementary Table S2. Complete search strategies

The final search was conducted on 25 December 2025. No language restrictions were applied. The PubMed search strategy was developed first and adapted for other databases according to database-specific syntax.

| Database | Records identified | Search strategy |
|---|---|---|
| PubMed | 151 | #1 "Lung Neoplasms"[Mesh] OR "lung neoplasm*"[tiab] OR "lung cancer*"[tiab] OR NSCLC[tiab] OR "non-small cell lung cancer"[tiab] OR "non small cell lung cancer"[tiab] OR "bronchogenic carcinoma*"[tiab]<br>#2 "Pneumonectomy"[Mesh] OR "Thoracic Surgery"[Mesh] OR "lobectomy*"[tiab] OR "segmentectomy*"[tiab] OR "pneumonectomy*"[tiab] OR "lung resection*"[tiab] OR "pulmonary resection*"[tiab] OR "thoracic surgery*"[tiab] OR VATS[tiab] OR thoracotomy*[tiab] OR thoracoscop*[tiab]<br>#3 "Exercise Therapy"[Mesh] OR "Breathing Exercises"[Mesh] OR "Respiratory Therapy"[Mesh] OR rehabilitation[Mesh] OR prehabilitation*[tiab] OR "preoperative rehabilitation*"[tiab] OR "pre-operative rehabilitation*"[tiab] OR "preoperative exercise*"[tiab] OR "preoperative training*"[tiab] OR "exercise training*"[tiab] OR "respiratory training*"[tiab] OR "breathing exercise*"[tiab] OR "inspiratory muscle training*"[tiab] OR "pulmonary rehabilitation*"[tiab]<br>#4 #1 AND #2 AND #3 |
| Web of Science | 401 | TS=("lung neoplasm*" OR "lung cancer*" OR NSCLC OR "non-small cell lung cancer" OR "non small cell lung cancer" OR "bronchogenic carcinoma*") AND TS=("lung resection*" OR "pulmonary resection*" OR lobectomy* OR segmentectomy* OR pneumonectomy* OR "thoracic surgery*" OR VATS* OR thoracotomy* OR thoracoscop*) AND TS=(prehabilitation* OR "preoperative rehabilitation*" OR "pre-operative rehabilitation*" OR "preoperative exercise*" OR "preoperative training*" OR "exercise training*" OR "respiratory training*" OR "breathing exercise*" OR "inspiratory muscle training*" OR "pulmonary rehabilitation*") |
| Embase (Ovid) | 22 | 1. exp lung tumor/ OR exp lung cancer/ OR ((lung adj3 (neoplasm* OR cancer* OR tumor* OR tumour* OR carcinoma*)).ti,ab.) OR NSCLC.ti,ab. OR "non-small cell lung cancer".ti,ab. OR "non small cell lung cancer".ti,ab. OR "bronchogenic carcinoma*".ti,ab.<br>2. exp lung resection/ OR exp pneumonectomy/ OR exp thorax surgery/ OR ("lung resection*" OR "pulmonary resection*" OR lobectomy* OR segmentectomy* OR pneumonectomy* OR "thoracic surgery*" OR VATS* OR thoracotomy* OR thoracoscop*).ti,ab.<br>3. exp exercise/ OR exp exercise therapy/ OR exp respiratory therapy/ OR exp breathing exercise/ OR exp rehabilitation/ OR (prehabilitation* OR "preoperative rehabilitation*" OR "pre-operative rehabilitation*" OR "preoperative exercise*" OR "preoperative training*" OR "exercise training*" OR "respiratory training*" OR "breathing exercise*" OR "inspiratory muscle training*" OR "pulmonary rehabilitation*").ti,ab.<br>4. 1 AND 2 AND 3 |
| Cochrane Library | 238 | #1 MeSH descriptor: [Lung Neoplasms] explode all trees<br>#2 ("lung neoplasm*" OR "lung cancer*" OR NSCLC OR "non-small cell lung cancer" OR "non small cell lung cancer" OR "bronchogenic carcinoma*"):ti,ab,kw<br>#3 #1 OR #2<br>#4 MeSH descriptor: [Pneumonectomy] explode all trees OR MeSH descriptor: [Thoracic Surgery] explode all trees<br>#5 ("lung resection*" OR "pulmonary resection*" OR lobectomy* OR segmentectomy* OR pneumonectomy* OR "thoracic surgery*" OR VATS* OR thoracotomy* OR thoracoscop*):ti,ab,kw<br>#6 #4 OR #5<br>#7 MeSH descriptor: [Exercise Therapy] explode all trees OR MeSH descriptor: [Breathing Exercises] explode all trees OR MeSH descriptor: [Respiratory Therapy] explode all trees OR MeSH descriptor: [Rehabilitation] explode all trees<br>#8 (prehabilitation* OR "preoperative rehabilitation*" OR "pre-operative rehabilitation*" OR "preoperative exercise*" OR "preoperative training*" OR "exercise training*" OR "respiratory training*" OR "breathing exercise*" OR "inspiratory muscle training*" OR "pulmonary rehabilitation*"):ti,ab,kw<br>#9 #7 OR #8<br>#10 #3 AND #6 AND #9 |
| CINAHL (EBSCOhost) | 22 | S1 (MH "Lung Neoplasms+") OR TI ("lung neoplasm*" OR "lung cancer*" OR NSCLC OR "non-small cell lung cancer" OR "non small cell lung cancer" OR "bronchogenic carcinoma*") OR AB ("lung neoplasm*" OR "lung cancer*" OR NSCLC OR "non-small cell lung cancer" OR "non small cell lung cancer" OR "bronchogenic carcinoma*")<br>S2 (MH "Thoracic Surgery+") OR (MH "Pneumonectomy+") OR TI ("lung resection*" OR "pulmonary resection*" OR lobectomy* OR segmentectomy* OR pneumonectomy* OR "thoracic surgery*" OR VATS* OR thoracotomy* OR thoracoscop*) OR AB ("lung resection*" OR "pulmonary resection*" OR lobectomy* OR segmentectomy* OR pneumonectomy* OR "thoracic surgery*" OR VATS* OR thoracotomy* OR thoracoscop*)<br>S3 (MH "Exercise Therapy+") OR (MH "Breathing Exercises+") OR (MH "Respiratory Therapy+") OR (MH "Rehabilitation+") OR TI (prehabilitation* OR "preoperative rehabilitation*" OR "pre-operative rehabilitation*" OR "preoperative exercise*" OR "preoperative training*" OR "exercise training*" OR "respiratory training*" OR "breathing exercise*" OR "inspiratory muscle training*" OR "pulmonary rehabilitation*") OR AB (prehabilitation* OR "preoperative rehabilitation*" OR "pre-operative rehabilitation*" OR "preoperative exercise*" OR "preoperative training*" OR "exercise training*" OR "respiratory training*" OR "breathing exercise*" OR "inspiratory muscle training*" OR "pulmonary rehabilitation*")<br>S4 S1 AND S2 AND S3 |
| CNKI | 240 | 主题 = (肺肿瘤 OR 肺癌 OR 非小细胞肺癌 OR 支气管肺癌) AND 主题 = (肺切除 OR 肺叶切除 OR 肺段切除 OR 全肺切除 OR 胸腔镜 OR 胸外科手术 OR 胸外科 OR |

| | | |
|---|---|---|
| | | VATS) AND 主题 = (预康复 OR 术前康复 OR 术前训练 OR 运动训练 OR 呼吸训练 OR 吸气肌训练 OR 肺康复 OR 呼吸功能训练) |
| Wanfang Data | 105 | 主题: (肺肿瘤 OR 肺癌 OR 非小细胞肺癌 OR 支气管肺癌) AND 主题: (肺切除 OR 肺叶切除 OR 肺段切除 OR 全肺切除 OR 胸腔镜 OR 胸外科手术 OR 胸外科 OR VATS) AND 主题: (预康复 OR 术前康复 OR 术前训练 OR 运动训练 OR 呼吸训练 OR 吸气肌训练 OR 肺康复 OR 呼吸功能训练) |
| VIP Database | 188 | 题名或关键词 = (肺肿瘤 OR 肺癌 OR 非小细胞肺癌 OR 支气管肺癌) AND 题名或关键词 = (肺切除 OR 肺叶切除 OR 肺段切除 OR 全肺切除 OR 胸腔镜 OR 胸外科手术 OR 胸外科 OR VATS) AND 题名或关键词 = (预康复 OR 术前康复 OR 术前训练 OR 运动训练 OR 呼吸训练 OR 吸气肌训练 OR 肺康复 OR 呼吸功能训练) |

## Supplementary Table S3. Sensitivity analyses

| Outcome | Studies (n) | Primary random-effects estimate | Fixed-effect estimate | Leave-one-out range | Interpretation |
|---|---|---|---|---|---|
| Overall PPCs | 13 | RR = 0.42, 95% CI 0.33-0.53; $I^2$ = 0% | RR = 0.42, 95% CI 0.33-0.53 | RR range 0.39-0.44; $I^2$ = 0% | Robust; direction and statistical significance unchanged. |
| Pneumonia or pulmonary infection | 12 | RR = 0.52, 95% CI 0.36-0.75; $I^2$ = 0% | RR = 0.52, 95% CI 0.36-0.75 | RR range 0.44-0.56; $I^2$ = 0% | Robust; direction and statistical significance unchanged. |
| Atelectasis | 15 | RR = 0.36, 95% CI 0.25-0.51; $I^2$ = 0% | RR = 0.36, 95% CI 0.25-0.51 | RR range 0.34-0.39; $I^2$ = 0% | Robust; direction and statistical significance unchanged. |
| Respiratory failure | 6 | RR = 0.37, 95% CI 0.17-0.78; $I^2$ = 0% | RR = 0.37, 95% CI 0.17-0.78 | RR range 0.28-0.40; $I^2$ = 0% | Robust, although based on a small number of events. |
| Length of hospital stay | 13 | MD = -2.22 days, 95% CI -2.52 to -1.92; $I^2$ = 52% | MD = -2.16 days, 95% CI -2.34 to -1.98 | MD range -2.31 to -2.11 days; $I^2$ range 19%-56% | Effect remained significant. Omitting Huang et al. reduced heterogeneity to $I^2$ = 22%, suggesting contribution to heterogeneity. |
| 6-minute walk distance | 4 | Not pooled because of substantial heterogeneity and clinical inconsistency | Not applicable | Not applicable | Synthesized narratively only. |

## Supplementary Table S4. Handling of multi-arm trials in the meta-analysis

Eligible intervention arms were combined at the trial-outcome level when they shared the same comparator and reported the same outcome. Continuous outcomes were combined using standard Cochrane methods; dichotomous outcomes were combined by summing events and sample sizes.

| **Study** | **Original trial arms** | **Outcome(s) and extracted data affected** | **Arm(s) included in meta-analysis / RevMan input** | **Handling method and rationale** |
|---|---|---|---|---|
| Gao et al. 2023 | C: cough and sputum training (n=60); I1: load-resistance respiratory muscle training for 5 days plus C (n=60); I2: same training for 10 days plus C (n=60). | Postoperative LOS: I1 7.62 +/- 2.18, I2 6.78 +/- 2.21, C 9.24 +/- 3.05 days. Chest-tube duration: I1 3.45 +/- 0.87, I2 3.03 +/- 0.76, C 4.18 +/- 1.03 days. Overall postoperative complications: I1 6/60, I2 5/60, C 10/60. | LOS was included using pooled I1 + I2 vs C: 7.20 +/- 2.23, n=120 vs 9.24 +/- 3.05, n=60. For chest-tube duration or overall postoperative complications, use pooled arms if synthesized: chest-tube duration 3.24 +/- 0.84, n=120 vs 4.18 +/- 1.03, n=60; overall complications 11/120 vs 10/60. | Pool eligible intervention arms. Both intervention arms met the intervention eligibility criteria and shared the same comparator; pooling avoids selecting only the more favorable 10-day arm. This study was not used for PPC-specific analyses because pulmonary complications were not reported separately. |

| Study | Original trial arms | Outcome(s) and extracted data affected | Arm(s) included in meta-analysis / RevMan input | Handling method and rationale |
|---|---|---|---|---|
| Huang et al. 2025 | HIIT group (n=30), aerobic training group (n=30), and control group (n=30). | LOS: HIIT 10.66 +/- 2.08, aerobic training 12.35 +/- 2.21, control 15.28 +/- 2.22 days. Postoperative infection was reported, but the infection type was not clearly specified as pneumonia or pulmonary infection. | Pool HIIT + aerobic training vs control for LOS: 11.51 +/- 2.29, n=60 vs 15.28 +/- 2.22, n=30. Postoperative infection was not included in the pneumonia/pulmonary infection meta-analysis. | Pool eligible intervention arms for LOS. Both HIIT and aerobic training were eligible exercise-based prehabilitation arms using the same control group; selecting HIIT alone could overestimate the intervention effect. The infection outcome was excluded from the pneumonia/pulmonary infection analysis because the endpoint definition was unclear. |
| Su et al. 2025 | CG: standard care (n=44); IG1: device-based respiratory training (n=44); IG2: respiratory training plus Liuzijue breathing exercise (n=44). | Postoperative pneumonia: IG1 8/44, IG2 8/44, CG 9/44. Length of hospital stay was reported as medians and interquartile ranges and was not pooled as a continuous mean-difference outcome. | Pool IG1 + IG2 vs CG for pneumonia/pulmonary infection: 16/88 vs 9/44. | Pool eligible intervention arms. Both IG1 and IG2 were preoperative respiratory training interventions and shared the same control group. |
| Fang et al. 2013 | A1: operable surgical patients receiving exercise and breathing training (n=22); A2: operable surgical control group (n=22); B: non-operative patients receiving training (n=17). | Surgical comparison data: overall PPCs 6/22 vs 9/22, atelectasis 4/22 vs 7/22, respiratory failure 4/22 vs 9/22, and LOS 11.8 +/- 3.23 vs 14.9 +/- 5.16 days for A1 vs A2. | Use A1 vs A2 only; exclude group B from postoperative surgical outcome meta-analyses. | No pooling. Group B did not undergo lung resection and was therefore not eligible for postoperative surgical outcome analyses. |
| Benzo et al. 2011 | Two separate randomized studies were reported in one publication. | PPCs, pneumonia/pulmonary infection, respiratory failure, LOS, and chest-tube duration where applicable. | Treat as separate trial records rather than as a single multi-arm trial. | Separate trial records. The publication reported two distinct RCTs rather than multiple arms sharing one control group. |

## Supplementary Table S5. Assessment of publication bias and small-study effects

| Outcome | Studies (n) | Funnel plot | Egger’s test | Qualitative assessment |
|---|---|---|---|---|
| Overall PPCs | 13 | Possible asymmetry (Supplementary Figure S1) | P = 0.016 | Possible small-study effects/publication bias; interpretation limited by small trials, single-center designs, and geographical clustering. |
| Pneumonia or pulmonary infection | 12 | No marked asymmetry (Supplementary Figure S2) | P = 0.065 | Egger’s test was suggestive despite no marked visual asymmetry; sparse events and geographical clustering may affect interpretation. |
| Atelectasis | 15 | No marked asymmetry (Supplementary Figure S3) | P = 0.625 | No statistical evidence of small-study effects; sparse events and small-study uncertainty remain possible. |
| Length of hospital stay | 13 | Mild asymmetry (Supplementary Figure S4) | P = 0.285 | Egger’s test did not provide statistical evidence of small-study effects; visual asymmetry may reflect clinical heterogeneity in perioperative pathways and intervention protocols. |
| Respiratory failure | 6 | Not assessed | Not performed | Publication bias could not be ruled out because fewer than 10 studies were available, events were sparse, and the evidence base was geographically clustered. |
| 6-minute walk distance | 4 | Not assessed | Not performed | Publication/reporting bias could not be ruled out because few studies reported this outcome, no pooled estimate was calculated, and the evidence base was geographically clustered. |

## Supplementary Table S6. GRADE certainty-of-evidence assessment

| Outcome | Overall certainty | Domains downgraded | Domain-specific judgments and rationale |
|---|---|---|---|
| Overall PPCs | Low | Risk of bias; publication bias | Risk of bias: serious, because most trials had some concerns, mainly related to randomization or selective reporting. Inconsistency: not serious ($I^2 = 0\%$). Indirectness: not serious. Imprecision: not serious (258 events; CI excluded no effect). Publication bias: serious, because funnel plot asymmetry and Egger's test suggested small-study effects (P = 0.016). |
| Pneumonia or pulmonary infection | Low | Risk of bias; publication bias | Risk of bias: serious, because most trials had some concerns. Inconsistency: not serious ($I^2 = 0\%$). Indirectness: not serious, although endpoint definitions varied across studies. Imprecision: not serious; CI excluded no effect. Publication bias: serious, because Egger's test was suggestive of small-study effects (P = 0.065), despite no marked funnel-plot asymmetry. |
| Atelectasis | Moderate | Risk of bias | Risk of bias: serious, because most trials had some concerns. Inconsistency: not serious ($I^2 = 0\%$). Indirectness: not serious. Imprecision: not serious; CI excluded no effect. Publication bias: not serious, because no marked funnel-plot asymmetry was observed and Egger's test did not indicate small-study effects (P = 0.625). |
| Respiratory failure | Low | Risk of bias; imprecision | Risk of bias: serious, because most trials had some concerns. Inconsistency: not serious ($I^2 = 0\%$). Indirectness: not serious. Imprecision: serious because events were sparse (26 total events) and the CI was wide. Publication bias: not downgraded because fewer than 10 studies were available and formal testing was not appropriate; this uncertainty was considered qualitatively. |

| | | | |
|---|---|---|---|
| Length of hospital stay | Low | Risk of bias; inconsistency | Risk of bias: serious, because most trials had some concerns. Inconsistency: serious, because moderate heterogeneity was observed ($I^2 = 52\%$). Indirectness: not serious, although discharge practices may be context-specific. Imprecision: not serious; CI was narrow and excluded no effect. Publication bias: not serious; visual asymmetry was considered, but Egger's test did not provide statistical evidence of small-study effects ($P = 0.285$). |
| Preoperative 6MWD | Very low | Risk of bias; inconsistency; imprecision; publication/reporting bias | Risk of bias: serious, because most trials had some concerns. Inconsistency: serious, because exploratory pooling showed substantial heterogeneity ($I^2 = 95\%$) and divergent directions of effect. Indirectness: not serious. Imprecision: serious, because only four studies with 274 participants were available and no pooled estimate was considered clinically appropriate. Publication/reporting bias: serious, because formal testing was not appropriate with only four studies and reporting bias could not be ruled out qualitatively. |

**Supplementary Figure S1. Funnel plot for overall postoperative pulmonary complications (PPCs).**

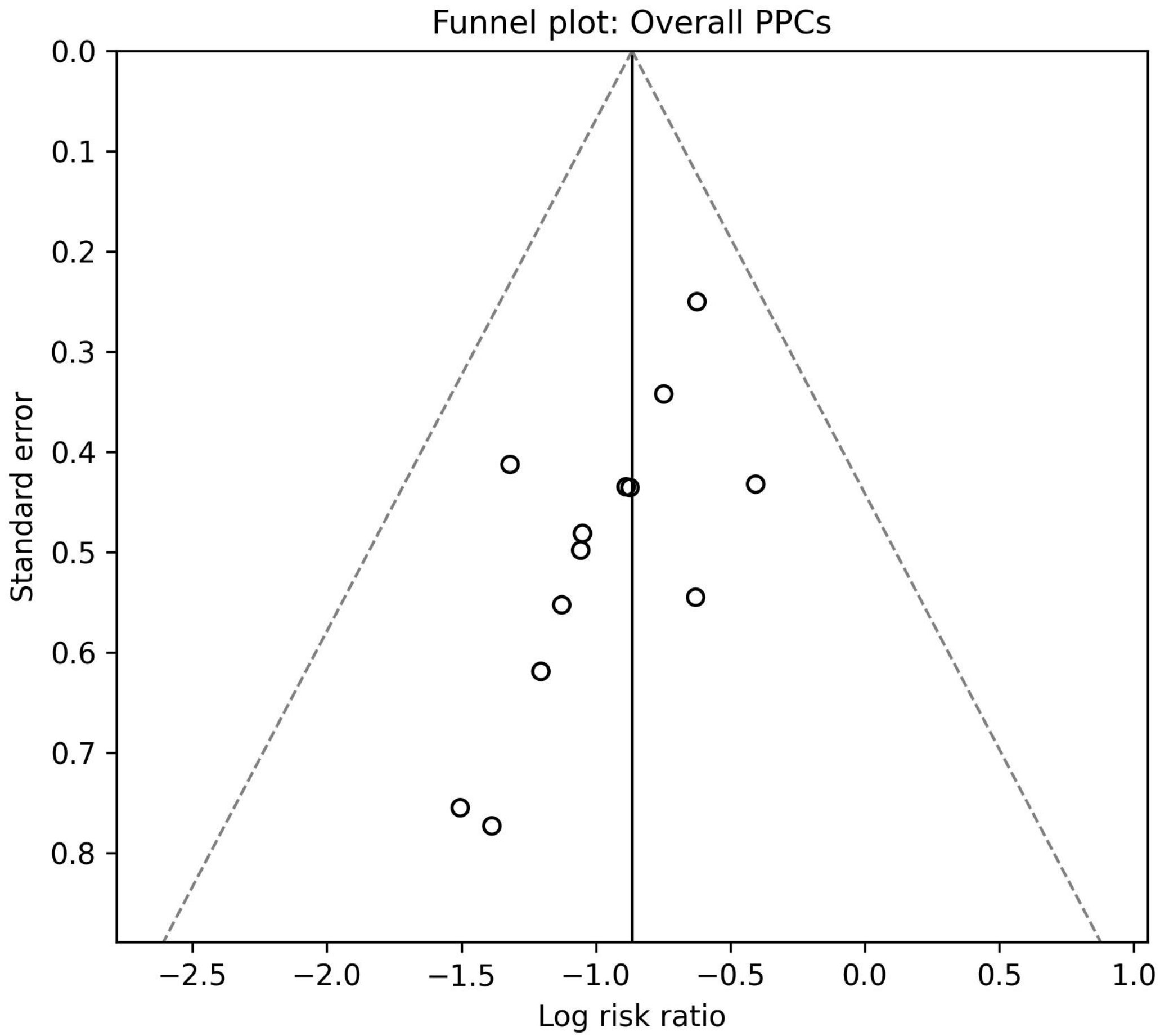

**Supplementary Figure S2. Funnel plot for pneumonia or pulmonary infection.**

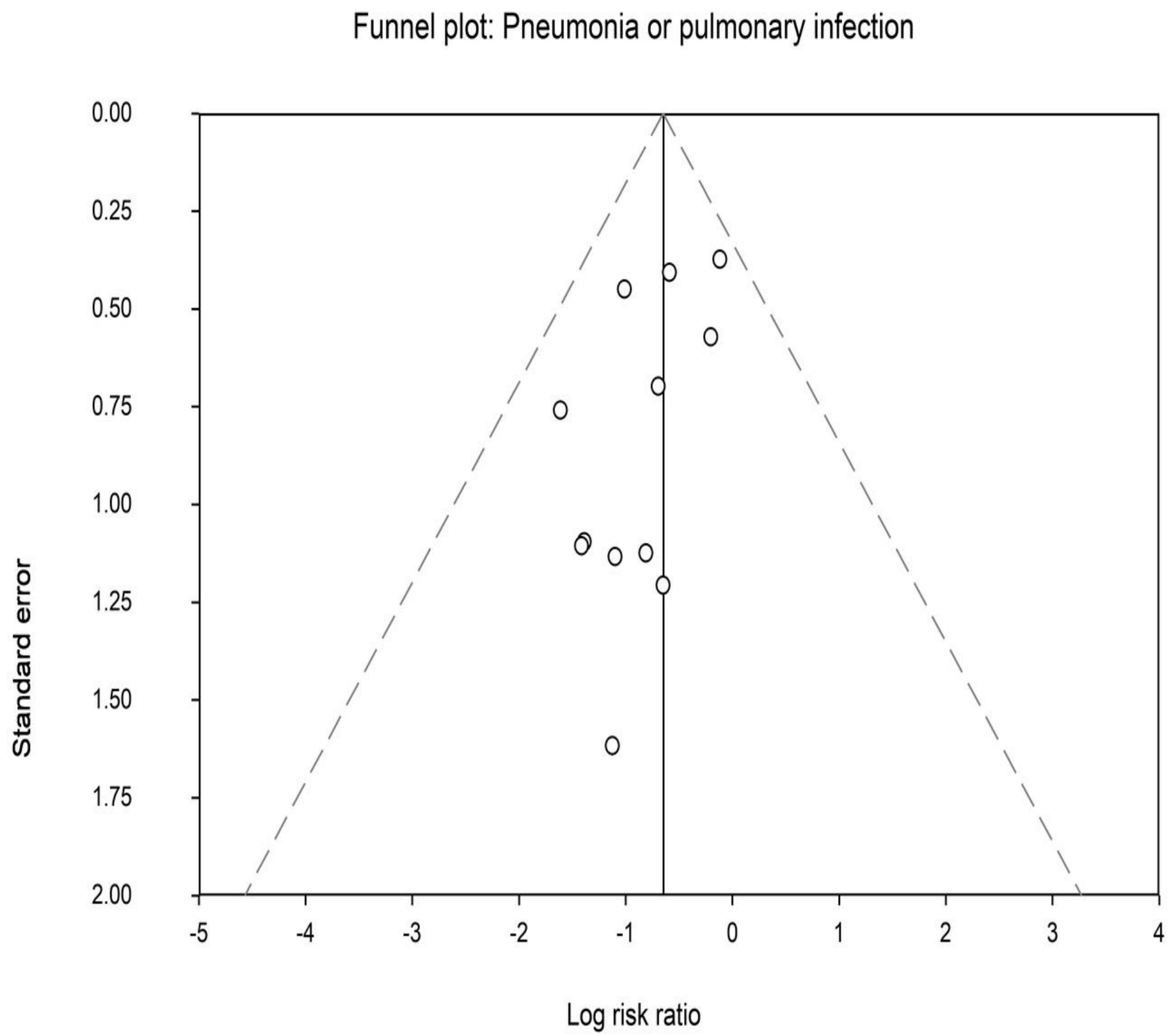

**Supplementary Figure S3. Funnel plot for atelectasis.**

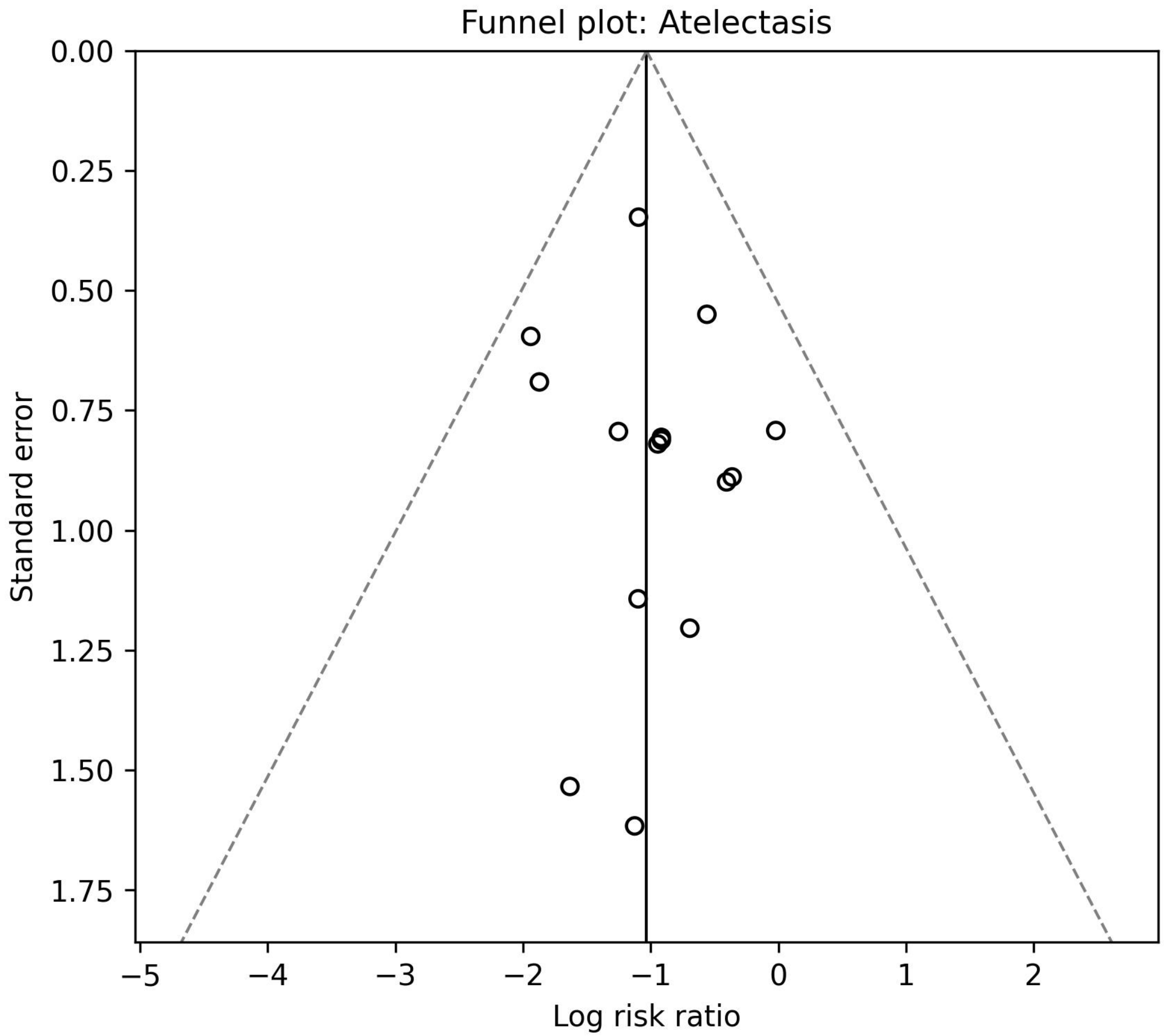

**Supplementary Figure S4. Funnel plot for postoperative length of hospital stay.**

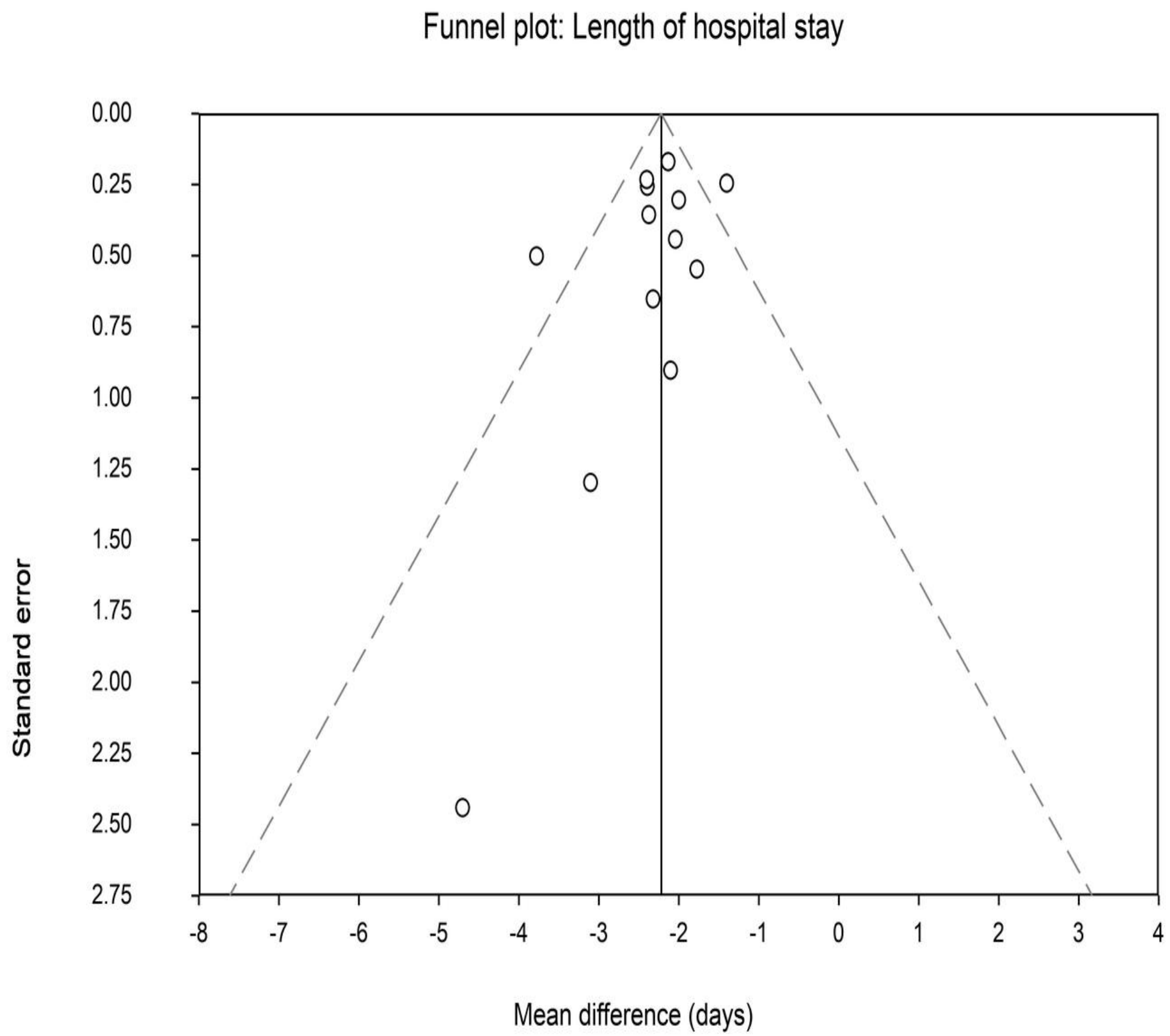

Supplement: Supplementary file 1 [file Data_Sheet_1.pdf]
